# Sociodemographic differences in dietary trends among Iranian adults: findings from the 2005–2016 Iran-WHO STEPS survey

**DOI:** 10.1017/S1368980023002203

**Published:** 2023-12

**Authors:** Sara Ebrahimi, Rebecca M Leech, Sarah A McNaughton, Farshad Farzadfar, Erfan Ghasemi, Sahar Saeedi Moghaddam, Katherine M Livingstone

**Affiliations:** 1 Deakin University, Institute for Physical Activity and Nutrition, School of Exercise and Nutrition Sciences, Geelong, VIC 3220, Australia; 2 Non-Communicable Diseases Research Center, Endocrinology and Metabolism Population Sciences Institute, Tehran University of Medical Sciences, Tehran, Iran; 3 Endocrinology and Metabolism Research Centre, Endocrinology and Metabolism Clinical Sciences Institute, Tehran University of Medical Sciences, Tehran, Iran

**Keywords:** Dietary trends, Iranian adults, Nutrition transition, WHO STEPS survey, Repeat-cross-sectional, Sociodemographic characteristics

## Abstract

**Objective:**

To examine trends in the intake of key food groups among Iranian adults between 2005 and 2016, overall, and according to sociodemographic characteristics.

**Design:**

Repeat cross-sectional data from the Iran-STEPwise approach to risk factor surveillance (Iran-WHO STEPS) 2005–2016 were analysed. Regression analyses were used to evaluate trends in the frequency of fruits, vegetables and fish intake and type of oil used over time. Interactions by sex, age and area of residence were examined.

**Setting:**

Iran.

**Participants:**

225 221 Iranian adults.

**Results:**

The frequency of vegetables (*β*: −0·03; 95 % CI (−0·06, −0·00); *P*-trend = 0·030) and fish (*β*: −0·09; 95 % CI (−0·10, −0·08); *P*-trend < 0·001) intake and use of solid fat (OR: 0·70; 95 % CI (0·70, 0·72); *P*-trend < 0·001) declined, whilst the frequency of fruit intake (*β*-Coeff: 0·03, 95 % CI (0·01, 0·05); *P*-trend = 0·014) and liquid oil use (OR: 1·40; 95 % CI (1·3, 1·4); *P*-trend<0·001) rose. Rising trends in fruit intake were larger in mid-aged (40–60 years) and older (>60 years) adults (*P*-interaction < 0·001), whilst declines in vegetable (*P*-interaction < 0·001) and fish intake (*P*-interaction = 0·001) were larger in older adults. The declining use of solid fat was strongest in middle-aged and older adults (*P*-interaction = 0·035), while the increasing use of liquid oil was strongest in rural areas (*P*-interaction = 0·011).

**Conclusions:**

During the nutrition transition, liquid oil use and the frequency of fruit intake rose, while the frequency of vegetables and fish intake declined. Nonetheless, the fatty acid composition and cooking methods are important considerations. The changes observed are concerning from a public health perspective and demonstrate the need for interventions and possible targets for tailored strategies.

A nutrition transition is a situation in which urbanisation, social development and the advent of modern technology result in changing quality and quantity of foods consumed. Specifically, high-quality foods such as fruits, vegetables and legumes are replaced by foods high in added sugars, fat and salt, animal-source foods and refined grains^(1,2)^. While a nutrition transition has been identified in developed countries including the United States and European countries over the past few decades, developing countries such as Indonesia and Myanmar are also experiencing changes in dietary intake^(2,3)^. The Eastern Mediterranean Region (EMR), which includes most Middle Eastern countries, has also experienced a nutrition transition as a result of urbanisation and technological development that has led to higher prevalence of chronic disease^(4)^.

The World Health Organization (WHO) has identified that Iran and neighbouring Arab countries located in the Middle East region, including Kuwait, Bahrain, Emirate, Qatar and Oman, have experienced a nutrition transition, in which dietary intake has changed towards increasing intake of foods high in saturated fat, added sugars and salt and lower intake of fruits and vegetables^(4–6)^. Iran is the second most highly populated country in the Middle East region with a population of over 80 million. It consists of a broad range of different ethnic groups, which are found in other Middle East countries such as Turks, Kurds and Arabs^(4,7,8)^. Since the 1970s, Iran has experienced economic development and rapid urbanisation, resulting in a reduction in infectious diseases and the advent of new challenges in rising rates of obesity and chronic diseases^(7,9)^. This economic and social development has played a significant role in changing dietary behaviours^(10)^. While limited studies investigated the type of oil used in this region, evidence shows that Iran and Arab countries have high intake of animal fat and saturated fat. This led the WHO to recommend that these countries apply a policy to reduce the intake of saturated fat in the region^(11,12)^. Findings report that Iranians use hydrogenated vegetable oils and solid fats for cooking and frying, baking breads and sweets due to their popular taste and aroma^(13)^. Moreover, although Iranian households have traditionally used animal fats for cooking^(13)^, government subsidisation of liquid oils is likely to have led to higher consumption of hydrogenated vegetable oils. Given the role of total fat and SFA in developing obesity and chronic diseases, it is essential to examine the use of different types of oils in the Iranian population^(14)^. It will also help to determine policy impacts on dietary trends^(15)^. Moreover, data from a national WHO study in 2011 suggest that up to 88 % of Iranian adults aged 15–64 years are consuming less than five servings of fruits and vegetables per day^(16)^.

Currently, only one study by Kimiagar *et al*.^(17)^ has examined trends in dietary intake in Iranian adults at the population level. In this repeat cross-sectional study of 5591 households, changes in the food intake of Iranian households were examined between 1963–1976 and 1991–1995. Results show that bread consumption declined, rice intake rose and trends in the consumption of dairy products did not change during these years. Conversely, there was a rise in the intake of fruits and vegetables, legumes, meats, eggs and total fats^(17)^. Evidence suggests that Iran has experienced a nutrition transition over the past five decades, however, no study has examined trends in food group intakes or use of specific oil types in the Iranian adult population after the year 2000^(4,7)^.

It is unclear whether the nutrition transition is disproportionately affecting different population groups in Iran. A repeat cross-sectional study conducted by Ghassemi *et al.* compared dietary intake trends between areas of residence (rural and urban areas) from 1985 to 1995^(7)^. This study showed that consumption of fruits and vegetables, dairy products and meats rose in rural areas, whilst they declined in urban areas^(7)^. Cross-sectional studies of Iranian adults suggest that dietary intakes vary by age^(18–20)^ and sex^(18–21)^, with higher intake of fruits in younger adults^(19,20)^ and in women^(18)^. However, no studies have examined differences in dietary trends according to age and sex and no studies have looked at overall trends and differences by population groups in the same study. Thus this study aimed to examine overall trends in dietary intake among adults between the years 2005 and 2016 and to investigate variations in these trends according to sociodemographic characteristics using data from the Iran-WHO STEPS 2005–2016^(22,23)^. This information will help identify which Iranian population groups, and in which regions, have shown worsening or improvements in dietary intake over the nutrition transition. Furthermore, most countries in the Middle East have experienced a nutrition transition and these countries have culturally specific diets that are characterised by high intake of hydrogenated fats^(24,25)^. Therefore, an understanding of trends in key food group intakes in the Middle East region, particularly Iran, is needed.

## Methods

### Subjects and study design

The Iran-WHO STEPS 2005–2016 is a repeat cross-sectional study that was conducted annually in Iran between 2005 and 2009, and then additionally in 2011 and 2016^(22,23)^. The WHO STEPS surveillance programmes aim to develop continuous evidence to inform the global prevention and control of non-communicable diseases^(22,23)^. The interviewers were trained on how to complete the questionnaire, and how to store and send the information^(26)^. Participants were included in the present analysis if they were aged 18 years or older. Participants were excluded if they had missing data for any sociodemographic characteristics (age, sex and area of residence) or food group intakes (fruit, vegetables, fish and type of oil)^(22,23)^. A cluster sampling method was used to recruit participants from all provinces of Iran between 2006 and 2009. In total, 50 clusters and 1000 individuals in each cluster were randomly selected from each province of Iran, while each cluster included 20 individuals, 10 male and 10 females^(22,23)^. A multistage cluster sampling method was used in 2005 and 2011. A total of 50 distinct counties were selected as the primary sampling units. Urban and rural areas were selected as secondary sampling units. The households were randomly selected using postal codes and one individual was recruited from each household^(27)^. A proportional-to-size cluster sampling method was used in 2016^(22,23)^. A total of 3105 clusters were selected from all provinces of Iran and 10 participants were recruited from each cluster^(27)^. Trained staff of universities of medical sciences in Iran collected self-reported information on dietary intake, sociodemographic characteristics, lifestyle behaviours and health characteristics. The range of response rate of the surveys was high throughout and ranged between 89·5 % in 2005 and 98·4 % in 2016^(22,23)^.

Participants were excluded from the present study if they were aged <18 years and had missing data for sociodemographic characteristics or food group intakes. A signed consent form was obtained from all participants. The Iran-WHO STEPS 2005 to 2011 was approved by the Iran Centre for Control of Diseases, and Iran-WHO STEPS 2016 was approved by the National Institute for Medical Research Development. The present analysis was granted an Ethics Exemption by Deakin University (reference number 2019-288). This manuscript is reported according to the Strengthening the Reporting of Observational Studies in Epidemiology—Nutritional Epidemiology reporting checklist (see online Supplemental Table 1)^(28)^.

### Study measures

#### Dietary intakes

Information on the intake of fruits, vegetables, fish and the use of type of oil was collected using a previously tested brief dietary questionnaire^(22,23)^. Data collected from open-ended questions included: the frequency of intake of fruits (d/week), vegetables (d/week) and fish (times/week). Participants were asked to select the type of oil that was usually used from the following options: solid fat, liquid oil, animal fat (ghee), margarine, no specific type of oil, never-use oil, any other oil, unknown and butter. Due to small numbers of responses (between 0·1 % and 0·3 %), butter was combined with animal fat (ghee) and unknown oil and other oils were combined with no specific type of oil. As a result, solid fat, liquid oil, animal fat, margarine, no specific oil and never-use oil were included in this analysis. There were some minor differences in the dietary questionnaires used between the years. For example, in 2005, fruit intake was assessed by asking participants how many days fruit was consumed in the last week, while in other years it asked how many days fruit was consumed in a typical week. A summary of the brief dietary questions is provided in Supplemental Table 2.

#### Sociodemographic characteristics

A self-report questionnaire was administered face-to-face by a trained interviewer to collect sociodemographic data including age, sex and area of residence. Participants were asked about their date of birth and selected their sex and area of residence from males and females and urban and rural areas, respectively. For the purpose of this study, age was categorised into three age groups including 18–≤40 years (young adults), 40–≤60 years (mid-aged adults), >60 years (older adults) based on previous research using the Iran-WHO STEPS^(29)^.

#### Data analysis

Data analyses were performed using weighted counts using the svy suite of commands for survey weighting (version 16.0; StataCorp.). A *P*-value of <0·05 was considered statistically significant. To address the differences between the Iran-WHO STEPS sample and the Iranian population, weights were calculated using sex and province according to the 2006 Iranian National Population and Housing Census^(30)^. The weighted mean and linearised standard errors were reported for continuous variables (fruit, vegetable and fish) and the weighted proportion of participants was reported for all categorical and binary variables (sociodemographic characteristics and type of oil). Linear regression analysis was used to evaluate the slopes (*β*-Coeff and 95 % CI) of frequency of fruit, vegetable and fish intake (d/week, times/week, dependent variables) over time (independent variable). The analysis was adjusted for age (continuous) and area of residence (binary). Each type of oil was treated as a binary outcome variable, and logistic regression analysis was used to evaluate the slopes (odds ratios (OR) and 95 % CI) in the type of oil used (dependent variable) over time (independent variable). Time was treated as an ordinal variable when reporting the mean and percentage of food groups in each year, and treated as a continuous variable when examining the P for trend for slopes across years. Interaction terms were added to the models to examine how trends in diet varied according to the following sociodemographic characteristics: age (categorical), sex (binary) and area of residence (binary). The overall significance of the interaction was determined using the testparm command. Interaction analyses were adjusted for age (continuous), sex (binary) and area of residence (binary), except when the given variable was used in the interaction term. For frequencies of fruit, vegetable and fish intake, the margins post hoc command was used to derive the marginal effect for each category of the sociodemographic characteristic over time using the dydx option. This command helps with identifying coefficients and *P* values for each sociodemographic category over time (e.g. females and males). For oil use, the lincom post hoc command was used to estimate the effect for each category of the sociodemographic characteristic over time.

## Results

**Fig. 1:**
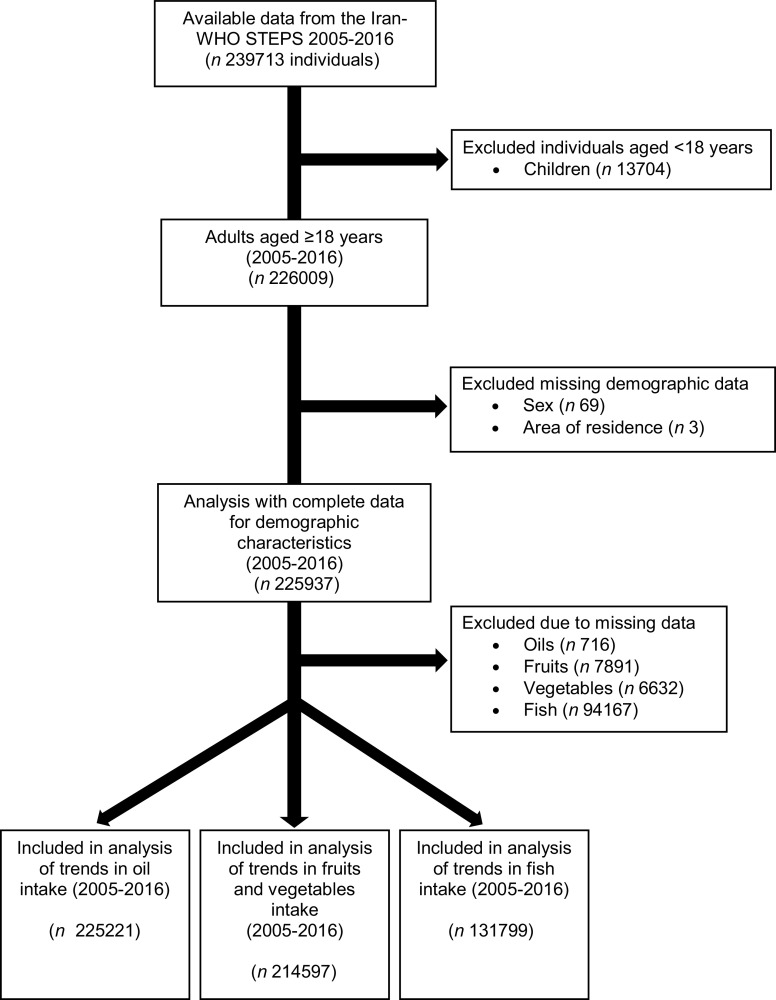
Participants flow diagram from the Iran-WHO STEPS surveys 2005–2016

Of the 239 713 individuals included in the WHO-STEP surveys, 13 776 individuals were excluded in the present analysis for being <18 years of age (*n* 13 704; 5·7 % participants excluded) and for missing data for sex (*n* 69; 0·03 % participants excluded) and area of residence (*n* 3; 0·001 % participants excluded). A total of 225 937 adults were included in the descriptive analysis of demographic characteristics. To minimise loss from missing data on food group intakes, three samples were used to examine trends in food groups between 2005 and 2016: fruit and vegetables (*n* 214 597; 5·02 % participants excluded), fish (*n* 131 799; 41·67 % participants excluded) and oil (*n* 225 221; 0·32 % participants excluded) (Fig. 1).

The weighted proportion of sociodemographic characteristics of Iranian adults between the years 2005 and 2016 is shown in Table 1. In all years, the majority of participants were younger aged males, and living in urban areas.

**Table 1:** Sociodemographic characteristics in Iranian adults from the Iran-WHO STEPS surveys 2005–2016 (*n* 225 937)

Sociodemographic characteristic*	2005	2006	2007	2008	2009	2011	2016
*n*	75 152	27 966	27 492	27 211	27 447	10 128	30 541
	%	%	%	%	%	%	%
Age categories†							
Younger aged	48·6	48·0	47·4	47·2	47·4	48·6	46·6
Mid-aged	43·7	43·1	43·3	43·3	43·4	34·7	35·3
Older aged	7·8	8·9	9·3	9·5	9·1	16·7	18·1
Sex							
Female	49·2	49·3	49·3	49·4	49·2	49·6	49·1
Male	50·8	50·7	50·7	50·6	50·8	50·4	50·9
Area of residence							
Urban	67·0	66·2	65·5	68·1	63·7	71·0	70·5
Rural	33·1	33·8	34·5	31·9	36·3	29·0	29·5

* Weighting proportions were estimated using sex and province weighting.

† Younger aged (18–≤40 years old), mid-aged (40–≤60 years old), older aged (>60 years old).

Trends in the intake of key food groups are presented in Table 2. Between 2005 and 2016, the frequency of intake of fruits (d/week) rose (*β*: 0·03; 95 % CI (0·01, 0·05); *P*-trend = 0·014), while there was a decline in the frequency of vegetables (d/week) consumed (*β*: −0·03; 95 % CI (−0·06, −0·00); *P* trend = 0·030), and the frequency of consumption of fish (times/week) (*β*: −0·09; 95 % CI (−0·10, −0·08); *P*-trend < 0·001) declined. Between 2005 and 2016, the proportion of participants who usually used solid fat declined from 79 % to 29 % (OR: 0·70; 95 % CI (0·70, 0·72); *P*-trend < 0·001), while the proportion of participants who used liquid oil, animal fat and no specific oil rose from 18 % to 64 % (OR: 1·40; 95 % CI (1·3, 1·4); *P*-trend < 0·001), 1·3 % to 3 % (OR: 1·13; 95 % CI (1·1, 1·2); *P*-trend < 0·001) and 0·7 % to 2 % (OR: 1·18; 95 % CI (1·1, 1·3); *P*-trend < 0·001), respectively. No significant trends were observed for margarine intake and never using oil.

**Table 2:** Trends in food group intakes and use of oils in Iranian adults from the Iran-WHO STEPS surveys 2005–2016

Food groups															Unadjusted			Adjusted		
	2005		2006		2007		2008		2009		2011		2016		Slope‡			Slope‡		
	Mean	se *	Mean	se *	Mean	se *	Mean	se *	Mean	se *	Mean	se *	Mean	se *	*β*-Coeff	95 % CI	*P* trend§	*β*-Coeff	95 % CI	*P* trend‖
Fruits (d/week)	4·5	0·17	3·8	0·18	4·1	0·17	4·3	0·15	4·1	0·16	4·5	0·18	4·3	0·20	0·03	0·01, 0·05	0·005	0·03	0·01, 0·05	0·014
Vegetables (d/week)	3·9	0·12	4·4	0·10	4·6	0·12	4·5	0·08	4·8	0·09	4·2	0·13	3·7	0·11	−0·03	−0·06, −0·00	0·039	−0·03	−0·06, −0·00	0·030
Fish (times/week)	1·7	0·08	0·58	0·10	1·4	0·12	1·7	0·10	1·7	0·12	0·81	0·11	0·47	0·06	−0·09	−0·11, −0·08	<0·001	−0·09	−0·10, −0·08	<0·001
Oils (%)†																				
Solid fat	79·5		70·9		58·4		48·2		48·9		38·6		29·8		0·71	0·70, 0·72	<0·001	0·70	0·7, 0·72	<0·001
Liquid oil	18·4		26·8		38·4		49·2		48·2		58·2		64·8		1·39	1·3, 1·4	<0·001	1·40	1·3, 1·4	<0·001
Animal fat	1·3		1·8		1·8		1·5		1·7		2·0		3·0		1·14	1·1, 1·2	<0·001	1·13	1·1, 1·2	<0·001
Margarine	0·07		0·03		0·03		0·03		0·02		0·06		0·08		1·08	0·9, 1·3	0·412	1·06	0·9, 1·3	0·511
No specific oil	0·65		0·39		1·38		0·96		1·21		0·96		2·0		1·19	1·1, 1·3	<0·001	1·18	1·1, 1·3	<0·001
Never use oil	0·07		0·05		0·09		0·10		0·05		0·12		0·05		1·01	0·89, 1·1	0·868	0·94	0·82, 1·1	0·401

* Unadjusted mean and SE.

† Unadjusted percentage.

‡ Slopes reflect *β*-Coeff and 95 % CI for fruits, vegetables and fish intake and odds-ratio and 95 % CI for use of oils.

§ Unadjusted *P* trend. Trends in fruits and vegetables (*n* pooled = 214 597; 2005 *n* 74 961; 2006 *n* 27 966; 2007 *n* 24 922; 2008 *n* 23 882; 2009 *n* 23 867; 2011 *n* 9844; 2016 *n* 29 155), and fish intake (*n* pooled = 131 799; 2005 *n* 34 152; 2006 *n* 27 964; 2007 *n* 12 638; 2008 *n* 9585; 2009 *n* 9034; 2011 *n* 8434; 2016 *n* 29 992), over time were examined by using Wald tests of associations for linear regression weighted for sex and province.

‖ *P* trend adjusted for age (continuous) and area of residence (binary). Trends in types of oil over time were examined by using Wald tests of associations for logistic regression (*n* pooled = 225 222; 2005 *n* 75 100; 2006 *n* 27 959; 2007 *n* 27 473; 2008 *n* 27 196; 2009 *n* 27 441; 2011 *n* 10 087; 2016 *n* 29 966), weighted for sex and province.

Trends in key food group intake by sociodemographic characteristics are presented in Supplemental Tables 3–5. Significant interactions were observed for trends in fruit, vegetables and fish intake by age only. While younger adults had non-significant trends in fruit intake between 2005 and 2016 (*β*: −0·02; *P*-trend = 0·157), mid-aged (*β*: 0·06; *P*-trend < 0·001) and older adults (*β*: 0·08; *P*-trend < 0·001) had an increase in fruit intake over time (*P*-interaction < 0·001). For vegetable intake, the younger (*β*: −0·05; *P*-trend = 0·006) and older aged (*β*: −0·07; *P*-trend = 0·001) adults had a decline in intake between 2005 and 2016, while trends were non-significant in the mid-aged adults (*β*: 0·00; *P*-trend = 0·926) (*P*-interaction < 0·001). Between 2005 and 2016, younger (*β*: −0·09; *P*-trend < 0·001), mid-aged (*β*: −0·08; *P*-trend < 0·001) and older adults (*β*: −0·11; *P*-trend < 0·001) had a decline in the frequency of fish intake (*P*-interaction = 0·001).

Trends in solid fat, no specific oils and never-use oil were significantly different between age groups and the trends in liquid oil, animal fat and never-use oil were significantly different between areas of residence (see online Supplemental Table 6). Between 2005 and 2016, younger (OR: 0·71; *P*-trend < 0·001), mid-aged (OR: 0·70; *P*-trend < 0·001) and older adults (OR: 0·70; *P*-trend < 0·001) had a decline in the use of solid fat (*P*-interaction = 0·035). Adults living in urban (OR: 1·38; *P*-trend < 0·001) and rural (OR: 1·45; *P*-trend < 0·001) areas had a rise in the use of liquid oil intake (*P*-interaction = 0·011). Furthermore, adults in urban (OR: 1·17; *P*-trend < 0·001) and rural (OR: 1·09; *P*-trend =0·002) areas had an increase in the use of animal fat (*P*-interaction = 0·042). Between 2005 and 2016, younger (OR: 1·16; *P*-trend = 0·001), mid-aged (OR: 1·18; *P*-trend < 0·001) and older adults (OR: 1·28; *P*-trend < 0·001) had a rise in the use of no specific oil (*P*-interaction = 0·012). Adults who lived in rural areas only (OR: 0·83; *P*-trend = 0·001) had a decline in never using oil (*P*-interaction = 0·026).

## Discussion

Many developed and developing countries have experienced a nutrition transition. Changes in dietary intake towards higher intake of sugars, saturated fat and lower intake of fruits and vegetables have taken place in Middle Eastern countries including Iran. This repeated analysis of the Iran-WHO STEPS cross-sectional surveys examined the 11-year trends in intake of key food groups, overall and by sociodemographic characteristics, in a nationally representative sample of 225 221 Iranian adults. To our knowledge, this is the first study that has evaluated trends in key food groups intakes in this population in the last two decades. The main findings were that the use of solid fat has declined considerably over time, while the use of liquid oil and animal fat has risen. These trends may reflect the impact of Iranian food policy approaches that have been implemented over the past two decades to reduce solid fat intake. Furthermore, although the frequency of fruit intake may have risen, the frequency of vegetable and fish intake declined. With the largest declines observed in older adults, these findings highlight the need for targeted interventions to improve dietary patterns in these age groups. While the frequency of fruit and vegetable intake was higher in females than males between 2005 and 2016, no significant differences were found in fruit and vegetable trends between females and males, which was in line with a previous study on Iran-WHO STEPS^(19)^.

This study showed that the trend in solid fat use has declined and almost halved between the years 2005 and 2016 while the use of liquid oil rose nearly three-fold. Based on the existing evidence, the average consumption of SFA and trans fatty acids in the EMR countries is over 10 % and 1 % of energy, respectively, which is above WHO recommended intakes. Regarding trans fatty acids intake, the EMR is among the highest intake regions with the highest SFA and trans fatty acids intake in Kuwait, Saudi Arabia, Egypt and Pakistan. As a result, the WHO issued a recommendation for the EMR to adopt initiatives to reduce intake of these fatty acids^(31)^. In addition, hydrogenated vegetable oils are also commonly used for cooking and frying in the EMR^(32)^. Findings report that Iranians use hydrogenated vegetable oils and solid fats for cooking and frying, baking breads and sweets^(13)^. Although the present study did not include data on different types of solid fat, previous research found that 12 % of calories consumed by Iranians in 2004 came from hydrogenated vegetable oil^(33)^. The observed trends in solid fat and liquid oil use are likely attributable to policy that was adopted in Iran in 2005 to shift solid fat intake towards the consumption of liquid oil^(33)^. Policy action has included increasing public awareness of the harmful effects of trans fatty acids via mass media campaigns, and collaboration with industry to limit the amount of trans fatty acids in edible oils to less than 10 % of calories, with the ultimate goal of trans fats less than 1 %^(33,34)^. Specifically, new industry regulation was introduced on oil fractioning technology to reduce trans fatty acid content, on palm oil imports to reduce palm oil use in the food industry and on food product labelling to show the trans fatty acid content and nutritional traffic light signposting^(33,35,36)^. Similar policies have been adopted in other Middle Eastern countries, where Saudi Arabia, Kuwait, Bahrain and Israel launched nutrition labelling on products. In addition, Saudi Arabia has implemented a regulation to reduce the trans fatty acids content of hydrogenated vegetable oil to 2 % of total fat^(37)^. However, it is worth mentioning that the benefits of liquid oil use vary depending on fatty acid composition and cooking methods. Frying liquid oils to high temperatures could result in the production of compounds with adverse effects on health^(38)^. Furthermore, animal fats consist of vitamins and essential fatty acids, which could be nutritionally beneficial^(39)^. Future research should investigate the fatty acid composition of liquid oil and cooking methods in the Iranian population.

Despite observed fluctuations in the trends in fruit and vegetable intake over the past two decades, the frequency of vegetable intake declined and the frequency of fruit intake slightly increased overall from 2005 to 2016. This provides supporting evidence for a nutrition transition in Iranian adults, although it conflicts with what studies found prior to the year 2000, when vegetable intake was increasing^(17)^. In line with our findings for differences by population groups, a previous study using Iran-WHO STEP data from 2007 to 2009 reported that daily consumption of fruits and vegetables was lower in older aged groups^(19)^. The reports identified low intake of fruits and vegetables (below 5 servings/d) in Middle Eastern countries, including Egypt, Kuwait and Saudi Arabia^(40,41)^.

The declining trend in vegetable intake observed in this study suggests that culturally appropriate dietary policies and interventions are needed to address any worsening of diets as a result of the nutrition transition in Iran. Improvements in modifiable risk factors, such as diet, are particularly critical to combat rising rates of obesity and chronic disease in Iran observed during the nutrition transition^(7)^. To address these challenges, pragmatic policies and interventions that aim to improve nutrition education and food literacy are needed, as well as programmes to support a healthy food environment.

This study found that the frequency of fish intake has declined in Iranian adults overall, while intake was slightly higher in mid-aged adults. Fish is not considered a common component of Iranian cuisine compared to other protein sources^(42)^, where chicken, mutton and beef are more commonly consumed^(43)^. Fish consumption varies between Middle Eastern countries, where countries such as the United Arab Emirates and Oman have the highest intake of seafood, and Syria and Iraq have the lowest intake of seafood^(44)^. The FAO reported that fish consumption has increased from 1 kg/capita in 1980 to 7·3 kg/capita in 2008 in Iran, however, it is still less than the world average (18·9 kg/capita in 2010)^(45)^. While reports from the FAO show an increase in fish consumption/capita in Iranians after 1980, the current findings show a decline in the trend of fish intake. As with any food groups examined in this study, any discrepancies with the wider literature may be explained by fluctuations in intake observed over a shorter period of time in the present study. Longer durations of follow-up may help determine whether the declining trends observed in this study persist. While fish intake is recommended by the Iranian Dietary Guidelines^(46)^, a long-term plan and reformed policy that includes nutrition education and public awareness campaigns, as well as strategies to improve the food supply system, may be needed to increase Iranian fish consumption^(43,47)^.

This study has several strengths. The repeated cross-sectional design facilitated the first examination of trends in key food group intake over the last two decades in Iran. The questionnaire used in this study has been used in the STEP survey in other countries, which enables comparison of results from Iran with other populations^(22)^. Furthermore, information on different types of oil was available in this study, enabling investigation of the impact of the Iranian policy to reduce the intake of solid fat. This study also included a large nationally representative sample of Iranian adults from all provinces of Iran, including both urban and rural areas. This enabled the examination of interactions by sociodemographic characteristics, which helped identify the characteristics of group of Iranians with suboptimal dietary intake. Furthermore, findings from this study are somewhat comparable to the Iranian Dietary Guidelines. For example, the Iranian Dietary Guidelines recommend consuming fruits three times every day, however, the results from this study showed that the frequency of consumption of fruits in Iranian adults is 4 d in a week, which is much less than the Iranian Dietary Guidelines.

Several limitations should be acknowledged. Firstly, the use of a brief dietary questionnaire meant that this questionnaire did not provide sufficient details to derive nutrient intakes. Furthermore, the questionnaire collected information on fats and oils via one summary question, which may have limited the understanding of how Iranians use fats for different purposes. Also, participants might have considered butter as a solid fat, resulting in fewer responses for butter. Secondly, the study design was repeated cross-sectional, which meant that different samples of individuals were included at each time point. This prohibited our ability to determine changes in individual intakes over time but allowed for population-level estimates and examination of trends. Thirdly, the questionnaire items used between 2005 and 2016 were not all framed in the same way, which might have introduced measurement differences and impacted the accuracy of the identified trends. Also, the dietary questionnaire has not been validated in this population and the proportion of missing data varied between food groups. Furthermore, the sampling method was different between the years 2005 and 2016, which might have impacted the results between different time points. For example, participants aged 15–64 years old were included between the years 2005 and 2011, while participants over 18 years old (with no upper age limit) were recruited in 2016. Thus, the representativeness of the sample in terms of age is likely to be lower in the previous years, which may also have impacted the examination of differences in terms of age groups. However, this study applied weighted counts to compare the study samples and the Iranian population. Fourthly, since Iran has experienced a nutrition transition, examining the trends in intakes of unfavourable food groups, such as fast food and soft drinks, might reveal additional important aspects of the nutrition transition in Iran, which were not available in this study. Fifthly, it should be considered that the Iranian Dietary Guidelines recommend consuming food groups such as legumes and lean meats and fish, which were not collected in the Iran-WHO STEP and thus not available for analysis in this study. Therefore, future research may benefit from examining the intake of food groups that are specifically recommended in the Iranian Dietary Guidelines. Future research should also quantify intakes to confirm that any trends in the frequency of intake also correspond to trends in amounts consumed. Sixthly, dietary intakes according to different ethnicities were not examined due to lack of data. Given Iran is an ethnically diverse country, providing information on changes in dietary intakes in different ethnicities would be helpful for furthering understanding of associations between diet and health outcomes in the Iranian population. Lastly, it is important to note that a comprehensive sanction has been imposed in Iran over the last two decades. This impacted a variety of financial sectors, including banking networks and oil sectors. Thus, this political climate has influenced the country’s economy. Future research is needed to investigate any possible effects of political and financial changes on Iranian dietary intake^(48)^.

### Conclusions

In this large repeated cross-sectional study of Iranian adults, we observed that solid fat use declined over time, while liquid oil and animal fat use increased. Furthermore, the intake of fruits rose slightly over time, particularly in mid-aged and older adults. In contrast, vegetables and fish intake declined, with the strongest declines in older adults. These findings provide supporting evidence for the impact of Iranian food policy approaches for decreasing solid fat use and the need for targeted dietary intervention to increase vegetable and fish intake among Iranian adults. Nonetheless, the fatty acid composition and cooking methods are important considerations when choosing and preparing vegetable oils. Future research is needed to examine trends in unfavourable food groups to understand the full impact of the nutrition transition in Iran.

## Supporting information

Ebrahimi et al. supplementary material 1 Ebrahimi et al. supplementary material

Ebrahimi et al. supplementary material 2 Ebrahimi et al. supplementary material

## Supplementary material

For supplementary material accompanying this paper visit https://doi.org/10.1017/S1368980023002203
