# Supplementary material for: Sociodemographic differences in dietary trends among Iranian adults: findings from the 2005–2016 Iran-WHO STEPS survey
Source: Public Health Nutr. 2023 Oct 20;26(12):2963–72. doi: 10.1017/S1368980023002203 (PMC10755385; doi:10.1017/S1368980023002203)
Supplement: Ebrahimi et al. supplementary material 2 — Ebrahimi et al. supplementary material [file S1368980023002203sup002.docx]

**Supplementary Table 1.** Strengthening the Reporting of Observational Studies in Epidemiology—Nutritional Epidemiology (STROBE-nut)

| **Item** | **Item nr** | **Reported on page #** |
| --- | --- | --- |
| **Title and abstract** | 1 | 1-2 |
| **Introduction** |  |  |
| Background, rationale | 2 | 3,4 |
| Objectives | 3 | 4 |
| **Methods** |  |  |
| Study design | 4 | 4,5 |
| Settings | 4 | 4 |
| Participants | 5 | 4 |
| Variables | 6 | 4,5 |
| Data sources - measurements | 7 | 4,5 |
| Bias | NA | NA |
| Study Size | 8 | 6 |
| Quantitative variables | 9 | 6 |
| Statistical Methods | 10 | 6 |
| **Results** |  |  |
| Participants | 11 | 7 And Figure 1 |
| Descriptive data | 12 | 7 and Table 1 |
| Outcome data | 13 | 7-8, Tables |
| Main results | 14 | 7-8 and Tables |
| Other analyses | NA | NA |
| **Discussion** |  |  |
| Key results | 15 | 8 |
| Limitation | 16 | 11 |
| Interpretation | 17 | 8-11 |
| Generalizability | 18 | 8-11 |
| **Other information** |  |  |
| Funding | 19 | Title page |
| Ethics | 20 | 4 |
| Supplementary material | 21 | Separate document |

| **Fruits** | | | **Vegetables** | | **Fish** | | **Oils** | |
| --- | --- | --- | --- | --- | --- | --- | --- | --- |
|  | **Question** | **Response** | **Question** | **Response** | **Question** | **Response** | **Question** | **Response** |
| **2005** | How many days in the last week did you consume fruits? | Days/week | How many days in the last week did you consume vegetables? | Days/week | How many times did you consume fish in the last week? | Times/week | What type of oils do you usually use? | Solid fat  Liquid oil  Animal fat  Butter  Margarine  Others  No specific oil  Never use oil |
| **2006-2009** | How many days in a typical week do you consume fruits? | Days/week | How many days in a typical week do you consume vegetables? | Days/week | How many times do you consume fish in a typical week? | Times/week | What type of oils do you usually use? | Solid fat  Liquid oil  Animal fat  Butter  Margarine  Others  No specific oil  Never use oil  Unknown |
| **2011** | How many days in a typical week do you consume fruits? | Days/week | How many days in a typical week do you consume vegetables? | Days/week | How many times do you consume fish in a typical week? | Times/week | What type of oils do you usually use? | Solid fat  Liquid oil  Animal fat  Butter  Margarine  No specific oil  Never use oil  Unknown |
| **2016** | How many days in a typical week do you consume fruits? | Days/week | How many days in a typical week do you consume vegetables? | Days/week | How many times did you consume fish in the last week? | Times/week | What type of oils do you usually use? | Solid fat  Liquid oil  Animal fat  Butter  Margarine  Others  No specific oil  Never use oil  Unknown |

**Supplementary Table 2**. Iran-WHO STEPS dietary intake questionnaires from 2005 to 2016

**Supplementary Table 3**. Trends in fruits intake in Iranian adults from the Iran-WHO STEPS surveys 2005-2016 according to age, sex, and area of residence

| **Fruits intake (days/week), mean (SE)**^1^ | **2005** | **2006** | **2007** | **2008** | **2009** | **2011** | **2016** | **Slope** ^2^ | **P-trend** | **P-interaction**^2^ |
| --- | --- | --- | --- | --- | --- | --- | --- | --- | --- | --- |
| **Age** |  |  |  |  |  |  |  |  |  | <0.001 |
| Younger aged (reference; n=103,447) | 4.8 (0.17) | 4.1 (0.20) | 4.2 (0.16) | 4.4 (0.14) | 4.2 (0.17) | 4.6 (0.18) | 4.4 (0.20) | -0.02 (-0.04, 0.01) | 0.157 |  |
| Mid-aged (n=89,708) | 4.4 (0.18) | 3.5 (0.17) | 3.9 (0.18) | 4.2 (0.16) | 3.9 (0.14) | 4.4 (0.19) | 4.4 (0.20) | 0.06 (0.04, 0.08) | <0.001 |  |
| Older age (n=21,441) | 4.2 (0.18) | 3.3 (0.18) | 3.9 (0.21) | 4.2 (0.22) | 3.7 (0.22) | 4.3 (0.19) | 4.2 (0.22) | 0.08 (0.05, 0.11) | <0.001 |  |
| **Sex** |  |  |  |  |  |  |  |  |  | 0.089 |
| Female (reference; n=108,634) | 4.6 (0.17) | 3.9 (0.18) | 4.2 (0.19) | 4.4 (0.17) | 4.2 (0.16) | 4.6 (0.18) | 4.4 (0.20) | 0.02 (0.00, 0.04) | 0.039 |  |
| Male  (n=105,962) | 4.5 (0.17) | 3.6 (0.18) | 3.9 (0.16) | 4.1 (0.13) | 3.9 (0.17) | 4.4 (0.18) | 4.3 (0.21) | 0.03 (0.01, 0.05) | 0.009 |  |
| **Area of residence** |  |  |  |  |  |  |  |  |  | 0.925 |
| Urban  (reference; n=137,646) | 4.9 (0.13) | 4.1 (0.14) | 4.4 (0.14) | 4.6 (0.12) | 4.4 (0.15) | 4.7 (0.16) | 4.6 (0.17) | 0.03 (0.00, 0.05) | 0.030 |  |
| Rural  (n=76,950) | 3.8 (0.18) | 3.2 (0.20) | 3.3 (0.14) | 3.6 (0.17) | 3.4 (0.15) | 3.9 (0.23) | 3.6 (0.20) | 0.03 (-0.00,0.06) | 0.071 |  |

^1^ Weighted unadjusted means and standard errors

^2^ Slopes represent β-coeff and 95% CI. Interaction terms were added to the regression models to examine how trends in diet varied according to age (categorical), sex (binary), and area of residence (binary). The overall significance of the interaction was determined using the testparm command. The interaction between time (continuous) and age (continuous) was adjusted for area of residence (binary); the interaction between time (continuous) and sex (binary) was adjusted for age (continuous) and area of residence (binary); the interaction between time (continuous) and area of residence (binary) was adjusted for age (continuous). The margins post-hoc command was used to derive the marginal effect for each category of the sociodemographic characteristic over time using the dydx option.

**Supplementary Table 4**. Trends in vegetables intake in Iranian adults from the Iran-WHO STEPS surveys 2005-2016 according to age, sex, and area of residence

| **Vegetables (days/week), mean (SE)^1^** | **2005** | **2006** | | **2007** | | **2008** | **2009** | **2011** | **2016** | **Slope**^2^ | **P-trend** | **P-interaction**^2^ |
| --- | --- | --- | --- | --- | --- | --- | --- | --- | --- | --- | --- | --- |
| **Age** |  | |  | |  |  |  |  |  |  |  | <0.001 |
| Younger aged, (reference; n=103,447) | 3.9 (0.12) | | 4.4 (0.10) | | 4.5 (0.11) | 4.5 (0.08) | 4.7 (0.10) | 4.10 (0.14) | 3.7 (0.11) | -0.05  (-0.08, -0.01) | 0.006 |  |
| Mid-aged (n=89,708) | 3.9 (0.12) | | 4.4 (0.10) | | 4.6 (0.13) | 4.6 (0.09) | 4.8 (0.09) | 4.4 (0.13) | 3.9 (0.11) | 0.00  (-0.03, 0.03) | 0.926 |  |
| Older age (n=21,441) | 3.7 (0.12) | | 4.3 (0.11) | | 4.5 (0.17) | 4.5 (0.09) | 4.6 (0.09) | 4.04 (0.13) | 3.6 (0.13) | -0.07  (-0.11, -0.03) | 0.001 |  |
| **Sex** |  | |  | |  |  |  |  |  |  |  | 0.369 |
| Female (reference; n=108,634) | 4.03 (0.12) | | 4.6 (0.10) | | 4.7 (0.13) | 4.7 (0.09) | 4.9 (0.10) | 4.4 (0.13) | 3.8 (0.11) | -0.04  (-0.07, -0.01) | 0.019 |  |
| Male (n=105,962) | 3.8 (0.12) | | 4.2 (0.09) | | 4.4 (0.11) | 4.4 (0.08) | 4.6 (0.09) | 3.9 (0.12) | 3.6 (0.10) | -0.03  (-0.06, -0.00) | 0.047 |  |
| **Area of residence** |  | |  | |  |  |  |  |  |  |  | 0.599 |
| Urban (reference; n=137,646) | 4.1 (0.12) | | 4.5 (0.11) | | 4.6 (0.10) | 4.6 (0.08) | 4.8 (0.08) | 4.3 (0.12) | 3.8 (0.10) | -0.04  (-0.06, -0.01) | 0.007 |  |
| Rural (n=76,950) | 3.6 (0.16) | | 4.2 (0.11) | | 4.4 (0.17) | 4.4 (0.12) | 4.7 (0.15) | 3.9 (0.18) | 3.4 (0.13) | -0.03  (-0.08, 0.02) | 0.246 |  |

^1^ Weighted unadjusted means and standard errors

^2^ Slopes represent β-coeff and 95% CI. Interaction terms were added to the regression models to examine how trends in diet varied according to age (categorical), sex (binary), and area of residence (binary). The overall significance of the interaction was determined using the testparm command. The interaction between time (continuous) and age (continuous) was adjusted for area of residence (binary); the interaction between time (continuous) and sex (binary) was adjusted for age (continuous) and area of residence (binary); the interaction between time (continuous) and area of residence (binary) was adjusted for age (continuous). The margins post-hoc command was used to derive the marginal effect for each category of the sociodemographic characteristic over time using the dydx option.

**Supplementary Table 5**. Trends in fish intake in Iranian adults from the Iran-WHO STEPS surveys 2005-2016 according to age, sex, and area of residence

| **Fish (times/week), mean (SE)^1^** | **2005** | **2006** | **2007** | **2008** | **2009** | **2011** | **2016** | **Slope**^2^ | **P-trend** | **P-interaction**^2^ |
| --- | --- | --- | --- | --- | --- | --- | --- | --- | --- | --- |
| **Age** |  |  |  |  |  |  |  |  |  | 0.001 |
| Younger aged (reference; n=64,870) | 1.7 (0.08) | 0.62 (0.10) | 1.4 (0.11) | 1.6 (0.10) | 1.6 (0.11) | 0.84 (0.11) | 0.47 (0.07) | -0.09  (-0.11, -0.08) | <0.001 |  |
| Mid-aged (n=52,848) | 1.7 (0.08) | 0.55 (0.10) | 1.4 (0.13) | 1.7 (0.11) | 1.7 (0.13) | 0.80 (0.11) | 0.48 (0.07) | -0.08  (-0.09, -0.07) | <0.001 |  |
| Older age (n=14,080) | 1.7 (0.11) | 0.52 (0.09) | 1.4 (0.18) | 1.6 (0.10) | 1.7 (0.15) | 0.72 (0.10) | 0.43 (0.06) | -0.11  (-0.13, -0.09) | <0.001 |  |
| **Sex** |  |  |  |  |  |  |  |  |  | 0.608 |
| Female (reference; n=66,619) | 1.7 (0.08) | 0.58 (0.10) | 1.4 (0.12) | 1.7 (0.10) | 1.7 (0.12) | 0.79 (0.11) | 0.46 (0.06) | -0.09  (-0.10, -0.08) | <0.001 |  |
| Male (n=65,179) | 1.7 (0.08) | 0.59 (0.10) | 1.4 (0.12) | 1.7 (0.10) | 1.6 (0.12) | 0.82 (0.11) | 0.47 (0.07) | -0.09  (-0.11, -0.08) | <0.001 |  |
| **Area of residence** |  |  |  |  |  |  |  |  |  | 0.316 |
| Urban (reference; n=86,467) | 1.6 (0.06) | 0.56 (0.09) | 1.4 (0.11) | 1.6 (0.08) | 1.5 (0.09) | 0.78 (0.09) | 0.47 (0.06) | -0.09  (-0.10, -0.08) | <0.001 |  |
| Rural (n=45,331) | 1.9 (0.13) | 0.62 (0.12) | 1.5 (0.17) | 1.9 (0.14) | 1.9 (0.17) | 0.88 (0.18) | 0.45 (0.09) | -0.10  (-0.12, -0.08) | <0.001 |  |

^1^ Weighted unadjusted means and standard errors

^2^ Slopes represent β-coeff and 95% CI. Interaction terms were added to the regression models to examine how trends in diet varied according to age (categorical), sex (binary), and area of residence (binary). The overall significance of the interaction was determined using the testparm command. The interaction between time (continuous) and age (continuous) was adjusted for area of residence (binary); the interaction between time (continuous) and sex (binary) was adjusted for age (continuous) and area of residence (binary); the interaction between time (continuous) and area of residence (binary) was adjusted for age (continuous). The margins post-hoc command was used to derive the marginal effect for each category of the sociodemographic characteristic over time using the dydx option.

**Supplementary Table 6**. Trends in oils intake in Iranian adults from the Iran-WHO STEPS surveys 2005-2016 according to age, sex, and area of residence^1^

| **Oils (%)** | **2005** | **2006** | **2007** | **2008** | **2009** | **2011** | **2016** | **Slope**^2^ | **P-trend^2^** | **P-interaction^2^** |
| --- | --- | --- | --- | --- | --- | --- | --- | --- | --- | --- |
| **Solid oil** |  |  |  |  |  |  |  |  |  |  |
| **Age** |  |  |  |  |  |  |  |  |  | 0.035 |
| Younger aged (reference;  n=107452) | 80 | 72 | 59 | 49 | 50 | 40 | 31 | 0.71  (0.69, 0.72) | <0.001 |  |
| Mid-aged (n=94784) | 79 | 70 | 58 | 48 | 48 | 37 | 29 | 0.70 (0.69,0.72) | <0.001 |  |
| Older age (n=22985) | 77 | 70 | 57 | 45 | 48 | 37 | 27 | 0.70 (0.69,0.72) | <0.001 |  |
| **Liquid oil** |  |  |  |  |  |  |  |  |  |  |
| **Area of residence** |  |  |  |  |  |  |  |  |  | 0.011 |
| Urban (reference;  n=142575) | 24 | 33 | 47 | 57 | 57 | 66 | 70 | 1.38  (1.36, 1.40) | <0.001 |  |
| Rural (n=82646) | 7.2 | 14 | 22 | 33 | 32 | 40 | 51 | 1.45 (1.40,1.50) | <0.001 |  |
| **Animal fat** |  |  |  |  |  |  |  |  |  |  |
| **Area of residence** |  |  |  |  |  |  |  |  |  | 0.042 |
| Urban (reference; n=142575) | 1.0 | 1.3 | 1.3 | 1.0 | 1.0 | 1.6 | 2.9 | 1.17  (1.1, 1.2) | <0.001 |  |
| Rural (n=82646) | 1.9 | 2.9 | 2.7 | 2.6 | 2.9 | 2.9 | 4.3 | 1.09  (1.03, 1.14) | 0.002 |  |
| **No specific oil** |  |  |  |  |  |  |  |  |  |  |
| **Age** |  |  |  |  |  |  |  |  |  | 0.012 |
| Younger aged (reference;  n=107452) | 0.56 | 0.51 | 1.1 | 0.90 | 1.2 | 0.90 | 1.6 | 1.16  (1.07, 1.26) | 0.001 |  |
| Mid-aged (n=94784) | 0.74 | 0.31 | 1.6 | 1.0 | 1.2 | 1.1 | 2.1 | 1.18  (1.1, 1.3) | <0.001 |  |
| Older age (n=22985) | 0.76 | 0.07 | 1.7 | 1.0 | 1.5 | 0.77 | 2.8 | 1.28  (1.13, 1.44) | <0.001 |  |
| **Never use oil** |  |  |  |  |  |  |  |  |  |  |
| **Age** |  |  |  |  |  |  |  |  |  | 0.0001 |
| Younger aged (reference; n=107452) | 0.03 | 0.01 | 0.01 | 0.04 | 0.02 | 0.02 | 0.03 | 0.99  (0.79, 1.26) | 0.996 |  |
| Mid-aged (n=94784) | 0.09 | 0.05 | 0.12 | 0.11 | 0.05 | 0.17 | 0.02 | 0.98  (0.85, 1.12) | 0.723 |  |
| Older age (n=22985) | 0.18 | 0.24 | 0.37 | 0.31 | 0.18 | 0.31 | 0.15 | 0.96  (0.83, 1.11) | 0.556 |  |
| **Area of residence** |  |  |  |  |  |  |  |  |  | 0.026 |
| Urban (reference;  n=142575) | 0.07 | 0.05 | 0.07 | 0.06 | 0.04 | 0.15 | 0.06 | 1.00  (0.86, 1.17) | 0.956 |  |
| Rural (n=82646) | 0.07 | 0.05 | 0.13 | 0.16 | 0.50 | 0.03 | 0.01 | 0.83  (0.74, 0.92) | 0.001 |  |

^1^ Data represent weighted unadjusted percentages; Only data from significant interactions are presented

^2^ Slopes represent Odds ratio and 95% CI. Interaction terms were added to the logistic regression models to examine how trends in diet varied according to age (categorical), sex (binary), and area of residence (binary). The overall significance of the interaction was determined using the testparm command. The interaction between time (continuous) and age (continuous) was adjusted for area of residence (binary); the interaction between time (continuous) and sex (binary) was adjusted for age (continuous) and area of residence (binary); the interaction between time (continuous) and area of residence (binary) was adjusted for age (continuous). The lincom command was used to derive the effect for each category of the sociodemographic characteristic over time.
