# Supplementary material for: Sociodemographic differences in dietary trends among Iranian adults: findings from the 2005–2016 Iran-WHO STEPS survey
Source: Public Health Nutr. 2023 Oct 20;26(12):2963–72. doi: 10.1017/S1368980023002203 (PMC10755385; doi:10.1017/S1368980023002203)
Supplement: Ebrahimi et al. supplementary material 1 — Ebrahimi et al. supplementary material [file S1368980023002203sup001.docx]

**Rachel Jones**

**Editor-in-Chief**

**Public Health Nutrition**

**23 February 2023**

Dear Professor Jones

We are pleased to submit for consideration for publication in **Public Health Nutrition**. This manuscript is a nationally representative study examining 11-year dietary trends in over 250,000 Iranian adults entitled “Sociodemographic differences in dietary trends among Iranian adults: findings from the 2005-2016 Iran-WHO STEPS survey”.

While Iran has experienced a nutrition transition over the last five decades, few studies have examined trends in dietary intake during this time. This study used repeated cross-sectional data from the Iran-WHO STEPwise approach to risk factor surveillance to examine trends in dietary intake between 2005 and 2016, overall, and according to sociodemographic characteristics. Our main findings were that the use of solid fat has declined over time, while the use of liquid oil and animal fat have risen, which align with policy action to reduce intake of solid fat during this period. Moreover, the frequency of vegetables and fish declined during this period. These findings provide important insights into how the nutrition transition has impacted on the diets of Middle Eastern populations and support the need for dietary interventions and policies to address any detrimental trends.

As the Corresponding Author, I am the guarantor for the manuscript and I confirm that I have had full access to all of the data in the study and take responsibility for the integrity of the data and the accuracy of the data analysis. The manuscript has not been published elsewhere, and, if accepted for publication in **Public Health Nutrition**, it will not be republished in any other journal in the same or similar form without the written consent of **Public Health Nutrition.** The manuscript has been read and approved by all authors and each author has contributed substantially to the design, analysis, preparation and discussion of the manuscript.

Yours sincerely

**Sara Ebrahimi**

Deakin University,

Institute for Physical Activity and Nutrition,

School of Exercise and Nutrition Sciences,

Melbourne, Melbourne Burwood Campus,

221 Burwood Highway, Victoria 3125, Australia

Tel: +61 416 440 406

Email: [s.ebrahimi@deakin.edu.au](mailto:s.ebrahimi@deakin.edu.au)
